# Sigma-1 Receptors in Depression: Mechanism and Therapeutic Development

**DOI:** 10.3389/fphar.2022.925879

**Published:** 2022-06-16

**Authors:** Peng Ren, Jingya Wang, Nanxi Li, Guangxiang Li, Hui Ma, Yongqi Zhao, Yunfeng Li

**Affiliations:** ^1^ Beijing Institute of Basic Medical Sciences, Beijing, China; ^2^ Department of Pharmaceutical Sciences, Beijng Institute of Radiation Medicine, Beijing, China; ^3^ State Key Laboratory of Toxicology and Medical Countermeasures, Beijing Key Laboratory of Neuropsychopharmacology, Beijing Institute of Pharmacology and Toxicology, Beijing, China

**Keywords:** sigma-1 receptors, depression, E/I balance, proteostasis, calcium

## Abstract

Depression is the most common type of neuropsychiatric illness and has increasingly become a major cause of disability. Unfortunately, the recent global pandemic of COVID-19 has dramatically increased the incidence of depression and has significantly increased the burden of mental health care worldwide. Since full remission of the clinical symptoms of depression has not been achieved with current treatments, there is a constant need to discover new compounds that meet the major clinical needs. Recently, the roles of sigma receptors, especially the sigma-1 receptor subtype, have attracted increasing attention as potential new targets and target-specific drugs due to their translocation property that produces a broad spectrum of biological functions. Even clinical first-line antidepressants with or without affinity for sigma-1 receptors have different pharmacological profiles. Thus, the regulatory role of sigma-1 receptors might be useful in treating these central nervous system (CNS) diseases. In addition, long-term mental stress disrupts the homeostasis in the CNS. In this review, we discuss the topical literature concerning sigma-1 receptor antidepressant mechanism of action in the regulation of intracellular proteostasis, calcium homeostasis and especially the dynamic Excitatory/Inhibitory (E/I) balance in the brain. Furthermore, based on these discoveries, we discuss sigma-1 receptor ligands with respect to their promise as targets for fast-onset action drugs in treating depression.

## Introduction

Mood disorders are the most common types of neuropsychiatric illness and are increasingly becoming a major cause of disability (Zhao et al., 2022). Among them, depression is a mainly persistent mood disorder that negatively impacts the social, vocational and educational aspects of people’s life (Chen et al., 2021). Furthermore, people suffering from long-term mental stress can develop mood disorders and they are especially vulnerable to cognitive impairment thus leading to a poor quality of life. Depression is a commonly occurring and recurrent mood disorder worldwide that is becoming a matter of global health (He et al., 2022). Unfortunately, the pandemic of COVID-19 in recent years has only aggravated those conditions (Zhang et al., 2020; Hampshire et al., 2021). Antidepressants are widely prescribed and act by increasing brain monoamine levels but they have a disappointingly low response rate (around 50%) and a significant lag period (4–6 weeks) in their activities when compared to placebos in clinical trials (de Vries et al., 2018). Thus, it is urgent to perform further research and develop new and effective antidepressants.

Sigma receptors are divided into two subtypes (sigma-1 and sigma-2 receptors) and recently, the sigma-1 receptor, a type of chaperonin (28 kD), has attracted increasing attention for its broad spectrum of biological functions and as a potential target for drugs treating many medical conditions. Extensive research has revealed that sigma receptors play pivotal roles in the etiology of CNS diseases, including Alzheimer’s disease (Maurice et al., 1998), Parkinson’s disease (Francardo et al., 2014), schizophrenia (Hashimoto, 2009a; Takizawa et al., 2009), Huntington’s disease (Bol'Shakova et al., 2017; Vetel et al., 2021), ischemic stroke (Urfer et al., 2014), drug addiction (Meunier et al., 2006), analgesia (Davis, 2015; Shin et al., 2020), depression (Kulkarni and Dhir, 2009; Salaciak and Pytka, 2022), anxiety (Kulkarni and Dhir, 2009; Wang et al., 2019; Salaciak and Pytka, 2022) and cognitive disorders (Salaciak and Pytka, 2022). Among them, mental disorders and cognitive deficits are currently the most widely studied areas. Agonist-associated ligands targeting the sigma receptor system have entered clinical trials (Ye et al., 2020) and have already been shown to be beneficial for patients suffering from these types of psychiatric disorders. Herein, based on preclinical studies, we discuss the functions and roles of sigma-1 receptors, mainly in depression, and we highlight the potential mechanism of action involved and therapeutic development as well as discussing future directions in this field.

### Sigma-1 Receptor Localization and Biologic Effects

The sigma-1 receptor is encoded by the sigma-1 gene and was initially classified as an opioid receptor subtype (Ye et al., 2020), but subsequent research has illuminated a vast and unique array of structural phenotypes and a unique amino acid sequence distinct from other mammalian transmembrane proteins (Kruse, 2017; Ossa et al., 2017). More information about the discovery history of sigma-1 receptors has been published (Ye et al., 2020). Sigma-1 receptors are resident proteins of the endoplasmic reticulum (ER) and are predominantly localized in the cholesterol-rich region of ER mitochondria-associated membranes (MAM) (Schmidt and Kruse, 2019; Voronin et al., 2020). This protein is identical in peripheral tissues and in the brain, and probably is similar in other tissues as well (Rousseaux and Greene, 2016; Schmidt and Kruse, 2019). In the brain, sigma-1 receptors are distributed most abundantly in the hippocampus and hypothalamus, followed by the cerebellar area, the dorsal raphe nucleus (DRN) and the locus coeruleus (LC) (Voronin et al., 2020; Salaciak and Pytka, 2022), and are mainly expressed in neurons and glial cells in the brain (Nguyen et al., 2015). These various sites are related to cognition and motor, emotion and endocrine functions, and are also further involved in the pathophysiology of psychiatric disturbances of depression (Voronin et al., 2020). In normal conditions, sigma-1 receptors are able to form Ca^2+^-sensitive complexes with the main ER chaperone binding immunoglobulin protein (Bip) (Weng et al., 2017; Schmidt and Kruse, 2019). However, specific activation of sigma-1 receptors, upon ligand-directed activation, leads to their separation from Bip, after which they can translocate to multiple cellular destinations, including mitochondrial membranes, nuclear membranes and plasma membranes to elicit their biological functions such as stabilizing IP3R, maintaining Ca^2+^ flow from the ER to mitochondria and producing ATP (Rousseaux and Greene, 2016; Kruse, 2017; Ossa et al., 2017; Weng et al., 2017; Schmidt and Kruse, 2019; Voronin et al., 2020; Ye et al., 2020).

### Sigma-1 Receptors Are Involved in Proteostasis and Calcium Homeostasis

Many aspects of proteostasis have been shown to be critical to normal cellular biological functions and disturbances in this homeostasis can lead to abnormal cellular functions, shorter cellular lifespans and can provide a pathological basis for disease development (Suhm et al., 2018). Proteostasis has been found to be closely involved in the pathogenesis of diseases such as depression, Huntington’s chorea, Parkinson’s disease, Alzheimer’s disease (Fornai and Puglisi-Allegra, 2021) and most cancers (Arpalahti et al., 2020). The abnormal aggregation of damaged proteins usually occurs in neurodegenerative diseases and other age-related disorders (Kurtishi et al., 2019; López-Otín and Kroemer, 2021). Therefore, maintaining the protein homeostasis of cells is essential to ensure normal brain conditions. Calcium ions are essential in maintaining normal cell biology, regulating cell membrane excitability by rapid depolarization, acting as secondary messengers and regulating protein activity and gene expression (Nicholls, 1986). In depression, calcium has been reported to be involved in neuroplasticity within neuronal circuits (Deutschenbaur et al., 2016), a significant role that was confirmed in a clinical trial (Bertone-Johnson et al., 2012). Available studies have revealed that sigma-1 receptors play an important role in the regulation of both types of homeostasis conditions. Herein we focused on the regulation of both types of homeostasis by sigma-1 receptors in depression.

### The Unfolded Protein Response (UPR)

Impaired cellular proteostasis contributes to the pathogenesis of depressive disorders (Ii and Dwivedi, 2019; Mao et al., 2019). Long term chronic emotional stress eventually leads to cellular stress, which is partly reflected in ER stress (Hayashi, 2015; Seo et al., 2017). The universal cellular response to ER stress is the activation of adaptation processes aimed at maintaining proteostasis (Hetz, 2012). Sigma-1 receptors have been shown to modulate the ER stress response and the subsequent UPR, which can influence protein stability and localization (Ho et al., 2018), through binding and modulating the ER stress response via the ER stress sensor inositol-requiring enzyme 1 (IRE1) (Rosen et al., 2019; Delprat et al., 2020). In fact, a large number of proteins that are included in the UPR are considered promising targets for pharmacological regulation in various deleterious conditions (Almanza et al., 2019).

In a series of animal behavioral experiments, increases in the expression of genes associated with the UPR and inflammation account for the pronounced depressive-like behaviors (Timberlake et al., 2019). In primary cultures of mouse hippocampal neurons, glutamate-dependent induction of the IRE1-XBP1 signaling pathway in distal dendrites facilitated the expression of brain-derived neurotrophic factor (BDNF) in the bodies of neurons. It has also been suggested that BDNF drives its own expression via activation of the PKA-IRE1-XBP1 cascade in dendrites thus regulating neurite development (Saito et al., 2018). Mori et al. (Mori et al., 2013), revealed that a sigma-1 receptor agonist stabilized IRE1 in the MAM region and activated the transcription factor X-box-binding protein 1 (XBP1), which may modulate BDNF expression and further regulate the anti-depressive effect. In regard to the UPR involvement in inflammation, sigma-1 receptors regulate IRE1 activity *in vivo*, in LPS-treated sigma-1 receptor knockout mice, and enhance IRE1 activation and the inflammatory response observed (Rosen et al., 2019).

Together, these data demonstrate the substantial contribution of UPR processes to the pathogenesis of depression. The agonistic effect on sigma-1 receptors ensures the regulation of ER stress sensors, the activation of transcription factors, the increased expression of the BDNF gene, anti-inflammation proteins and chaperones. The combination of these processes probably contributes to the survival of neurons in target areas of the brain and the development of antidepressant action.

### Sigma-1 Receptors Directly Modulate the Biophysical Properties of Ca^2+^ Ion Channels

Sigma-1 receptors have been shown to associate with and directly regulate voltage-gated ion channels (VGICs) that belong to all superfamilies (Na^+^, K^+^ and Ca^2+^) and ionotropic glutamate receptors (NMDARs) (Aishwarya et al., 2021). These considerations make Sigma-1 receptors powerful and pluripotent regulators of neuronal activity, from synaptic transmission to intrinsic excitability. Thus, sigma-1 receptors may have great significance in the regulation of Ca^2+^-dependent mechanisms of antidepressant action.

It is well known that Ca^2+^ controls neuronal activity and plays an important role in many use-dependent forms of neuroplasticity induced by BDNF and glutamatergic mechanisms (Catterall, 2010; Baydyuk et al., 2015). Sigma-1 receptors were reported to modulate the intracellular Ca^2+^ concentration through both the regulation of membrane voltage-gated Ca^2+^ channels and Ca^2+^ mobilization from endoplasmic stores (Monnet, 2005; Su et al., 2010). Tchedre et al. (Tchedre et al., 2008) showed that sigma-1 receptor activation with (C)-SKF-10047 inhibits Ca^2+^ currents, while that effect was reversed by a sigma-1 receptor antagonist BD-1047, which appears to be mediated directly through sigma-1 receptor binding to L-type voltage-gated Ca^2+^ channels. Consistent with these findings, sigma-1 receptors also inhibit store-operated Ca^2+^ entry by diminishing the coupling of stromal interaction molecule 1 (STIM1) to calcium release-activated calcium channel protein 1 (Orai1) (Srivats et al., 2016). Besides voltage-gated Ca^2+^ channels, sigma-1 receptors also regulate non-voltage-gated Ca^2+^-permeable channels via direct protein-protein interactions (PPI), including IP3 receptors at the ER level and plasma membrane acid-sensing ion channels 1a (ASIC1a) (Mari et al., 2015).

In an animal behavior model, Ca^2+^-dependent mechanisms have been proven to be involved in the mechanism of the antidepressant effect. Urani et al. (Urani et al., 2002) reported that EGTA, a Ca^2+^ chelator, when administered to Swiss mice 10 min before a forced swim test (FST), had no effect on their immobilization time, but abolished the antidepressant-like action of igmesine (a selective sigma-1 receptor agonist) in a dose-dependent manner. Similarly, verapamil, a Ca^2+^ channel blocker, had a comparable effect (Urani et al., 2002). In fact, intracellular Ca^2+^ modulators also play an important role in the action of igmesine; more information is summarized in (Guo et al., 2020).

**FIGURE 1:**
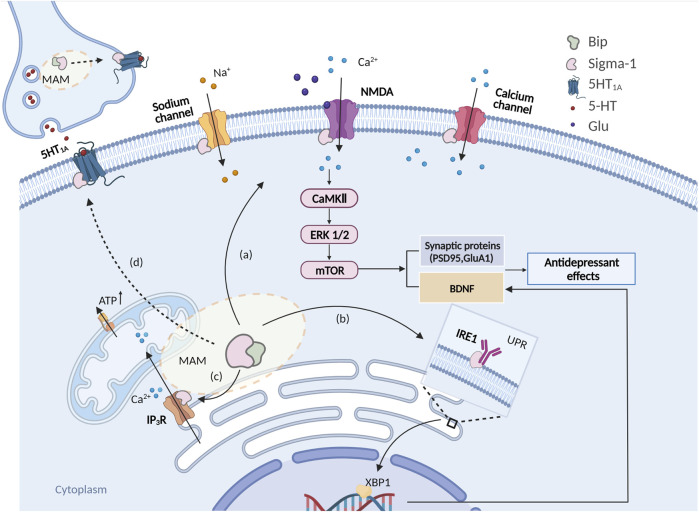
Schema of intracellular signaling pathways involved in the antidepressant-like effects of sigma-1 receptors. **(A)** Sigma-1 receptors are activated and translocated to the plasma membrane to interact with the NMDAR of pyramidal cells, which would result in a rapid intracellular activation of CaMKII that would eventually activate (phosphorylate) intermediates in the ERK1/2 and mTOR pathways, thus inducing a rapid synthesis of PSD95, BDNF etc; **(B)** Sigma-1 receptors are activated and then disassociate from Bip thus stabilizing IP3R, maintaining the Ca^2+^ flow from the ER to mitochondria and ATP production; **(C)** Sigma-1 receptor activates and thus binds and modulates the ER stress response via ER stress sensors IRE1 and facilitates BDNF expression via the IRE1-XBP1 signaling pathway. **(D)** Sigma-1 receptors may also modulate 5HT1A function through sigma-1 receptor-5HT1A interaction.

When it comes to the Ca^2+^ signal pathway, Calmodulin-dependent protein kinases (CaMKs) are inevitably involved. Among them, CaMKIV and CaMKII have been intensively studied and are involved in the transcription factor CREB-mediated BDNF signal pathway (Voronin et al., 2020). CaMKIV/II is an intracellular Ca^2+^-sensitive sensor. High concentrations of Ca^2+^ activate CaMK IV/II, and then eventually activate (phosphorylate) intermediates in the ERK1/2 and mTOR pathways (Cabanu et al., 2022), thus inducing a rapid synthesis of PSD95 as well as facilitating the phosphorylation of CREB, thus boosting the expression of BDNF involved in neuroplasticity (Fukunaga and Moriguchi, 2017) (Figure 1). Moriguchi et al. (Moriguchi et al., 2015) used a CaMK deficiency strategy *in vivo* to reveal that the mechanism of antidepressant action of sigma-1 receptors may be due to regulation of the intracellular Ca^2+^ level and activation of an alternative CaMKII-dependent mechanism for controlling the expression of BDNF. Moreover, chronic administration of fluoxetine and paroxetine to CaMKIV^−/−^ mice did not cause a pronounced antidepressant-like effect or the induction of neurogenesis in the hippocampal dentate gyrus (Moriguchi et al., 2015). In contrast, administration of the selective agonist SA4503 or fluvoxamine (with a high affinity for Sigma-1 receptor) for 2 weeks caused a decrease in the immobilization time of CaMKIV^−/−^ mice in the FST and the tail suspension test (TST), while those effects were abolished by preliminary treatment with the sigma-1 receptor antagonist NE-100 (Moriguchi et al., 2015). The abovementioned evidence indicates that a significant role of sigma-1 receptors in regulating Ca^2+^-dependent mechanisms of antidepressant action may not only be related to extracellular Ca^2+^ influx but also to intracellular Ca^2+^ homeostasis (Urani et al., 2002; Choi et al., 2022).

## Sigma-1 Receptors Regulate Excitatory and Inhibitory Balance

The classic “monoamine hypothesis” underlies the development of most clinical antidepressants that primarily exert their effects by enhancing the function of monoamine transmitters (Li, 2020). The translocation property of sigma-1 receptors allows them to modulate proteins directly not only at the ER-mitochondrion interface but also at the membrane where many ion channels, receptors and kinases are found, rendering sigma-1 receptors a unique inter-organelle signaling modulator in living tissues, including the CNS that we focused on (Su et al., 2010). In recent years, an increasing body of evidence suggests that sigma-1 receptors directly interact with proteins at neuronal cell membranes (more information is reviewed in (Ryskamp et al., 2019)) and enhance neurotransmission (Bermack and Debonnel, 2001; Bermack and Debonnel, 2005; Sambo et al., 2017; Sambo et al., 2018). The active roles of sigma-1 receptors in the regulation of neurotransmission, including the glutamatergic, GABAergic, serotonergic, dopaminergic (Sambo et al., 2018) and noradrenergic systems (Dhir A. and Kulkarni SK., 2008), is well documented. Those neurotransmission systems may form the basis for the dynamic balance of E/I neural networks in the brain. Especially, sigma-1 receptors have been reported to regulate presynaptic glutamate release and modulate NMDA receptor activity via direct PPI associations (Kourrich, 2017). Furthermore, sigma-1 receptor activation can also lead to alterations in NMDA receptors that upregulate and traffic to the plasma membrane thus further modulating neuronal intrinsic excitability (Pabba et al., 2014). In addition, Mtchedlishvil et al. found that pregnenolone and a selective sigma-1 receptor agonist (SKF-10047) inhibit the GABA-dependent inhibitory postsynaptic currents in rat hippocampal cell cultures (Mtchedlishvili and Kapur, 2003). We therefore suggest that sigma-1 receptors may regulate the E/I balance in direct or indirect manners. Importantly, there is abundant evidence that the medial prefrontal cortex (mPFC) or the hippocampus rely on the dynamic balance between E/I neurotransmitters for various advanced functions, such as emotion regulation and expression, as well as cognitive functions. Those two neurotransmitters achieve a dynamic balance to maintain normal physiological function under normal circumstances (Ferguson and Gao, 2018). Preclinical studies have shown that chronic stress can lead to decreased E/I neurotransmitter transmission in the mPFC (Fee et al., 2017). Consistent with this, a recent study found that chronic stress can lead to decreased glutamate and GABA neurotransmitter transmission in rat mPFC (Fee et al., 2017; Duman et al., 2019). In this context, GABAergic and glutamatergic neurotransmission may be almost rebalanced but the synapses remain impaired, thus the E/I rebalance may be at a low level. However, we recently found in our laboratory that regulation of the E/I rebalance in the mPFC may be an important mechanism and rate-limiting step in the efficacy of antidepressant effects (Yin et al., 2021).

The glutamatergic and GABAergic as well as serotonergic systems are heavily implicated in antidepressant actions. In this section, we mainly focused on the possible mechanisms by which sigma-1 receptors exert their antidepressant effects through the regulation of E/I balance by theseneurotransmitter systems.

### Sigma-1 Receptors and Glutamatergic Neurotransmission

Cortical excitability reflects a balance between E/I. Glutamate is the main excitatory neurotransmitter in the mammalian cortex (Petroff, 2002). Glutamate receptors have been pharmacologically classified as ionotropic and metabotropic receptors. Ionotropic glutamate receptors include the N-methyl-d-aspartic acid (NMDA) receptor (GluNs/NMDAr, GluAs), α-amino-3-hydroxy-5-methylisoxazole-4-propionic acid (AMPA) and kinase families of receptors. In this review, we will focus on NMDA and AMPA receptors due to their close link to depression and/or antidepressant action (Bermack and Debonnel, 2005). It appears that glutamatergic neurotransmission is altered during depressive episodes (Sanacora et al., 2003; Bermack and Debonnel, 2005; Duman et al., 2019). In addition, a clinical study revealed that glutamate metabolism differed significantly between depressed patients and controls (Paul and Skolnick, 2003), while those differences can be resolved with chronic antidepressant treatments (Mauri et al., 1998).

Similarly, numerous studies have shown interactions between sigma receptor and NMDA-receptor mediated responses in neurotransmission. The regulation of sigma-1 receptors with GluN1 can be explained by the PPI (Balasuriya et al., 2013) and an increase in its phosphorylation by protein kinases A and C (PK A/C) under sigma-1 receptor ligand activation of the chaperone protein (Kim et al., 2008). Moreover, Pabba et al. (Pabba et al., 2014) reported that a robust upregulation of GluN2A and GluN2B subunit expression was observed after treatment with the sigma-1 receptor agonist pentazocine (PTZ) or SKF-10074 injection and chronic administration of BD1047 prevented that effect. Conversely, a decreased protein level of GluN1 in the prefrontal cortex, hippocampus and amygdala was observed in a depression rat model while a two-week administration of SA4503 caused an anti-depressive-like effect, accompanied by a restoration of the GluN1 level (Wang et al., 2007).

Furthermore, in a sigma-1 receptor function deficient mouse model, sigma-1 receptor knockout (KO) mice are characterized by an inhibition of neurite outgrowth and impaired GluN2b function in the hippocampal dentate gyrus (Sha et al., 2013). Conversely, a study by Snyder et al. (Snyder et al., 2016), reported that compared to wild-type (WT) mice, AMPA receptor and NMDA receptors were unaffected in sigma-1 receptor KO mice. However, in regard to NMDA receptor-dependent long-term potentiation (LTP) and neuronal plasticity, sigma-1 receptor KO mice showed a mild deficiency (Snyder et al., 2016; Zhang et al., 2017). Interestingly, sigma-1 receptor agonists increased the expression of GluN2A and GluN2B subunits and postsynaptic density protein 95 (PSD-95), which is required for synaptic plasticity associated with NMDA receptor signaling (Pabba et al., 2014). Thus, sigma-1 receptors may play an important role in NMDA-receptor mediated functions, e.g., depression and cognitive disorder. On the other hand, sigma-1 receptors involved in a “long feedback loop” participated in the NMDA-receptor mediated antidepressant effect. Sustained treatments with sigma-1 ligands lead to a potentiation of NMDA-receptor-mediated responses in the mPFC and/or other brain regions, which in turn could lead to the modulation of serotonergic neurotransmission in the DRN (Peyron et al., 1998; Bermack and Debonnel, 2005). Overall, sigma-1 receptors have been indicated to be involved in the mechanism of action of antidepressants via the regulation of glutamatergic neurotransmission.

### Sigma-1 Receptors and GABAergic Neurotransmission

**FIGURE 2:**
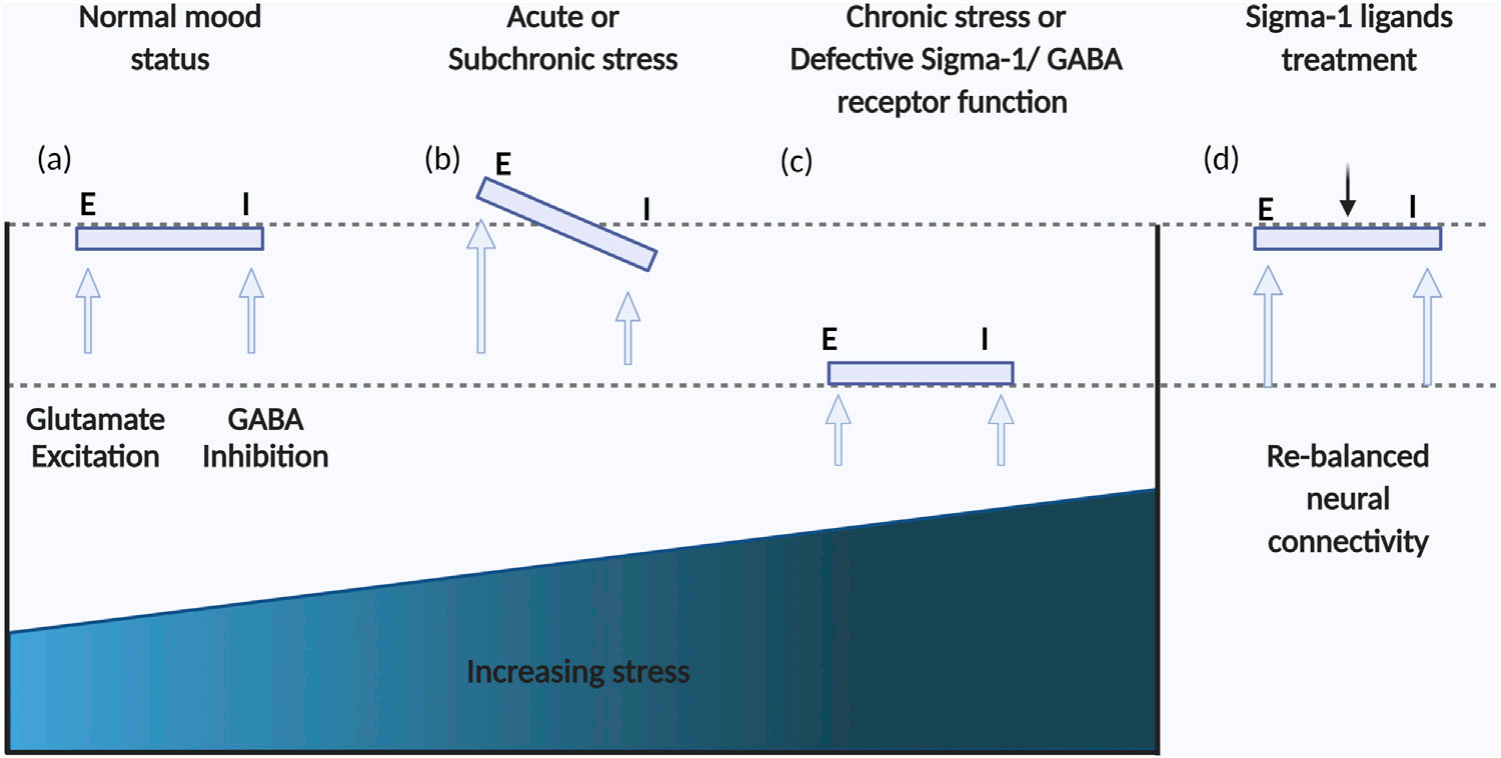
Schema of the dynamic balance of GABAergic and glutamatergic neurotransmission. **(A)** In a normal mood status, GABAergic and glutamatergic neurotransmission are balanced; **(B)** Acute stress can result in imbalances between GABAergic and glutamatergic neurotransmission; **(C)** Further adaptation to chronic stress may result in a new balance of GABAergic and glutamatergic neurotransmission but at lower levels, contributing to depression; **(D)** Regulating 5-HT-Glu/GABA long neural circuit through treatment with sigma-1 receptor ligands has the potential to rapidly restore the primary balance and level.

Chronic stress induced emotional disorders such as anxiety and depression involve imbalances between the excitatory glutamatergic system and the inhibitory GABAergic system in the PFC, GABA being the main inhibitory neurotransmitter in the mature mammalian CNS (Page and Coutellier, 2019) (Figure 2). Moreover, the majority of data on GABAergic deficiencies in depression have been gathered and demonstrated by means of indirect/direct methods, such as assessments of GABA levels in cerebrospinal fluid (CSF), brain specimens obtained post-mortem, by brain imaging, or by other pharmacological studies (Della et al., 2021). There is currently little compelling evidence that any sigma receptor(s) interacts with GABA receptors directly *in vivo*. Recent evidence has revealed that sigma-1 receptors modulate GABA uptake, transport-mediated release and exocytosis. Interestingly, a sigma-1 receptor antagonist decreased glutamate release but induced a biphasic response for GABA, while low doses of NE-100 increased GABA uptake, and with increasing doses, the uptake rate decreased (Pozdnyakova et al., 2020). Neurosteroids are a class of endogenous steroids that have potent effects on GABA receptors. In a circulating neurosteroid deficient rat model, Ago et al. (Ago et al., 2016) suggested that interactions between brain 5-HT1A and sigma-1 receptors may contribute to the treatment of GABA_A_ receptor deficit-related psychiatric disorders. On the other hand, a sigma-1 receptor deficiency reduces GABAergic inhibition in the basolateral amygdala leading to long term depression (LTD) impairment and depressive-like behaviors (Zhang et al., 2017).

Importantly, sigma-1 receptors may be involved in the “long feedback loop” that projects from the dorsal DRN to the mPFC and back to the DRN (Bermack and Debonnel, 2005). Hypidone hydrochloride (YL-0919) is a new antidepressant with a novel chemical structure that was developed by our laboratory, which has been found to have a high affinity for sigma-1 receptors (unpublished data). A recent study suggested that YL-0919 preferentially inhibits GABAergic neurons and reduces inhibitory input to pyramidal neurons, and that 5-HT1A receptor participates in the inhibition of GABA neurons thus regulating the E/I balance related to depression (Zhang et al., 2021). In general, inhibition of the spontaneous release of GABA may facilitate the release of other neurotransmitters throughout the CNS, thus altering the functions of other neurotransmitter systems. However, further study is essential to investigate whether sigma receptors are involved in this regulation in a direct and/or indirect manner.

### Effect of Sigma-1 Receptors on Serotonergic Neurotransmission

When it comes to antidepressant effects, the 5-HT system is necessarily involved. It is well known that 5-HT plays a key role in depression and/or the mechanism of action of antidepressants (Liu et al., 2017; Miquel-Rio et al., 2022). Both animal behavioral experiments and electro-physiological studies revealed that sigma-1 receptors increase 5-HT neurotransmission exerting antidepressant effects through various mechanisms. In a behavioral model, progesterone and BD-1047 (a sigma-1 receptor antagonist) counteracted the antidepressant-like effect induced by co-administration of pramipexole and sertraline (Rogoz and Skuza, 2006). In addition, in cell assays, classical antidepressants (fluvoxamine, etc.) significantly potentiated the NGF-induced neurite outgrowth, and the effects of all these drugs were antagonized by NE-100. Furthermore, the similar effects of mirtazapine were abolished by the 5-HT1A receptor antagonist WAY-100635 (Ishima et al., 2014). In another behavioral study, the sigma receptor ligand EMD- 57445 did not affect several 5-HT related parameters such as 8-OH-DPAT-induced behavioral syndrome or L-5-hydroxytryptophan-induced head twitches (Skuza et al., 1997). Thus, the effects of sigma-1 receptors on the 5-HT system seem to be controversial and fortunately electrophysiological experiments on 5-HT neurons have given us more direct evidence that sigma-1 receptors enhance serotonergic neurotransmission in a rapid manner (Robichaud and Debonnel, 2004; Lucas et al., 2008).

Previous studies have shown that acute and short-term treatments with SSRIs lead to a decreased firing activity of 5-HT DRN neurons, while long-term treatments lead to the restoration of 5-HT firing activity and this phenomenon explains the delayed onset of the action of SSRIs (Le Poul et al., 2000). Based on this model, Bermack and Debonnel assessed the effects of sigma-1 receptor ligands on the firing activity of 5-HT neurons in the DRN using an electrophysiological model with *in vivo* extracellular recording. Their study provided direct evidence that sigma-1 receptors are involved in serotonergic neurotransmission (Bermack and Debonnel, 2001). In that study, an increase in firing rates of 5-HT neurons of the DRN was observed after short term (2-days) or long-term (21-days) treatments with (+)-pentazocine, while those effects were completely abolished by co-administration of NE-100 (10 mg/kg/day) (Bermack and Debonnel, 2001). Similar evidence was found in another study (Lucas et al., 2008), where a 2-days continuous treatment with SA-4503 (1–40 mg/kg/day) increased the 5-HT neuron firing rate in a dose-dependent manner. Moreover, the firing rate of pyramidal neurons was recorded in the CA3 subfield of the hippocampus in further studies, the results suggesting that WAY100635 had a clear excitatory action in rats receiving chronic treatment of SA- 4,503 (10 mg/kg/day) for 2 days (Lucas et al., 2008; Maurice, 2021). Thus, facilitation of the 5-HT neuron firing rate induced by 2-days treatment with SA-4503 translated into the appearance of a 5-HT1A-mediated tonic inhibitory effect on CA3 pyramidal neurons, which may be involved in a 5-HT-Glu/GABA long neural circuit. In this way, sigma-1 receptors may be involved in modulating 5-HT neuronal activity and the E/I balance by 5-HT transporter combined with some receptors such as 5-HT1A (Li, 2020).

Importantly, these experiments show that sigma-1 receptor ligands have the potential to work as antidepressants with a rapid onset of action, due to an increase in 5-HT neuron firing activity after drug administration in just 2 days, a more rapid and robust anti-depressive effect than the majority of known antidepressant medications (Bermack and Debonnel, 2001).

### Sigma-1 Receptors and Other Neurotransmission System

In addition to the three neurotransmitter systems mentioned above, sigma-1 receptors also act on other neurotransmitter systems in the brain, including dopamine (DA) neurotransmission, noradrenaline (NE) neurotransmission and acetyl choline (Ach) neurotransmission (Katz et al., 2016). Among them, sigma-1 receptors modulate depression by acting on dopamine neurotransmitters is widely reported. For instance, PTZ enhances the antidepressant activity of the dopamine reuptake inhibitor bupropion while sigma-1 receptor antagonist reversed the effects (Dhir A. and Kulkarni S., 2008). Likewise, ropinirole (a D2/3 dopamine receptor agonist), elicited a significant anti-immobility effect in FST or FST, and the reduced immobility time exhibited by ropinirole attenuated by a sigma-1 receptor antagonist (Dhir and Kulkarni, 2007). These results reflect the regulatory role of sigma-1 receptor on the dopaminergic system in the brain. However, researchers have not intensively explored the potential mechanism of this unexpected results. Fortunately, the results of studies in recent years on sigma-1 receptors and DA neurotransmission in other disease models may help us to solve this confusion. Borroto-Escuela et al. (Borroto-Escuela et al., 2017) reported that cocaine self-administration induced a selective and significant increase in the density of D2R-sigma-1receptor positive clusters in the nucleus accumbens shell. Furthermore, the formation of the D2R-sigma-1 receptor heterodimer enhanced the ability of acute cocaine to increase the function of the D2R protomer and significantly reduced its internalization (Borroto-Escuela et al., 2019). In fact, receptor-receptor interactions in heterogeneous receptor complexes are widely present in CNS and are involved in the regulation of a variety of neuropsychiatric dysfunctions (Borroto-Escuela et al., 2020). Especially, the translocator property of the sigma-1 receptors gives it the opportunity to form heteroreceptor complexes with a variety of receptors to enhance the function of the original receptors, and this partly explains why sigma-1 receptor antagonists can block many of the unexpected effects aforementioned. The sigma-1 receptor complexs appear to hold the highest promise, it not only provides a new vision for our future research, but also a new strategy for the treatment of depression.

## Sigma Receptor Ligand Development for Treating Clinical Depression

Sigma-1 receptors are recently explored targets for treating depression and anxiety. Many antidepressants that are currently marketed are known to act through the sigma-1 receptor pathway (Hashimoto, 2009b). Furthermore, some novel compounds based on the sigma-1 receptor confirmation are being synthesized and tested in depressive animal models, more information reviewed in (Salaciak and Pytka, 2022). Although the exact therapeutic contribution of sigma-1 receptor binding remains to be unraveled, available data suggests that the anti-depressive efficacy is partly ascribed to sigma-1 receptor modulation. Some of the ongoing or completed clinical studies of sigma receptors are listed in Table 1 *.* Herein, we focus on clinical advances in the treatment of depression with sigma-1 receptor ligands.

**TABLE 1:** Sigma-1 receptor agonists in clinical studies.

Sigma-1 Agonist	Conditions	Clinic phase	ClinicalTrials.gov identifier
SA-4503	Major depressive disorder	Phase 2 unreleased	NCT00551109 (2007)
	Acute ischemic stroke	Completed	NCT00639249 (2008)
Pridopidine	Huntington disease	Phase 3 recruiting	NCT04556656 (2020)
	levodopa-induced dyskinesia	Phase 2 recruiting	NCT03922711 (2019)
	Huntington’s disease	Completed	NCT00724048 (2008)
	Huntington’s disease	Completed	NCT00665223 (2008)
ANAVEX2-73	Alzheimer’s disease	Phase 2b/3 recruiting	NCT04314934 (2020) NCT03790709 (2018)
	Rett syndrome	Phase 2 recruiting	NCT04304482 (2020) NCT03941444 (2019)
	Parkinson’s disease with dementia	Phase 2 recruiting	NCT03774459 (2018)
	Mild to moderate Alzheimer’s disease	Phase 2 active	NCT02756858 (2016)
	Alzheimer’s disease	Completed	NCT02244541 (2014)
T-817MA (Edonerpic Maleate)	Mild to moderate Alzheimer’s disease	Phase 2 completed	NCT02079909 (2014) NCT00663936 (2008)
	Mild cognitive impairment	Phase 2 recruiting	NCT04191486 (2019)
Igmesine	Major depression	Completed	https://doi.org/10.1016/S0924-977X(99)80011-X (1999)

Igmesine (JO-1784), one of the first discovered sigma-1 receptor ligands, was investigated in a clinical study of major depression in 1999 (Pande et al., 1999). Igmesine (25 mg/day) showed a statistically significant superiority over the placebo in the outpatient group, however, the compound ultimately failed to be effective in phase III clinical trials (Pande et al., 1999). SA-4503 (Cutamesine) is an orally available, potent and highly selective sigma-1 receptor agonist. Cutamesine not only decreased the immobility time in the FST but also played an anti-depressive-like behavior in an olfactory bulbectomized rat model of depression. In 2007, a phase II clinical study of SA4503 was performed, where SA4503 was given once daily for 8 weeks and then tested to determine safety and efficacy in 150 subjects with major depression. However, the trial data and outcome summaries have yet to be released (NCT00551109).

The sum of these results suggests that sigma-1 receptors affect the release of various neurotransmitter systems that have been shown to be involved in the pathophysiology of depression. We conclude that sigma-1 receptor agonists may have an antidepressant activity and are expected to be effective drugs for treating depression in the future. However, inconclusive results from different clinical trials have led to setbacks in the further development of these molecules for the treatment of depression.

## Discussion and Conclusion

Depression is a common mental disorder that affects approximately 300 million people worldwide (Zhao et al., 2022). Even worse, according to a scientific brief that was recently released by the World Health Organization (WHO), the COVID-19 pandemic triggered a 25% increase in the prevalence of anxiety and depression globally, which poses a significant challenge to mental health care (Who, 2022). Classical “monoamine strategy” drugs mostly target a series of defects in clinical applications. Therefore, there is an increasing interest in investigating modern monoamine (optimized multi-targets) strategies with faster-acting and fewer side-effects. In this way, sigma-1 receptors have entered the limelight, with their translocation property allowing them to modulate proteins directly.

To date, at least 49 proteins with highly divergent sequences and structures have been reported to interact with sigma-1 receptors. Due to the complicated nature of their effects on the downstream signaling pathways, it is not surprising that sigma-1 receptors play a role in maintaining the balance of glutamatergic, GABAergic, serotonergic, noradrenergic, and dopaminergic systems in the brain (Katz et al., 2016). As mentioned earlier, *in vivo* electrophysiological recordings revealed that sigma-1 receptor agonists, such as (+)-pentazocine or SA-4503, markedly increased 5-HT neuron firing after 2 or 21 days of treatment while a selective sigma-1 receptor antagonist, NE-100, blocked those effects. In addition, SA-4503 at 10 mg/kg/day induced the appearance of a 5-HT1A-mediated inhibitory tonus on hippocampal pyramidal neurons, as revealed by intravenous injections of the selective 5-HT1A antagonist WAY100635. In fact, recent findings from our laboratory revealed a similar phenomenon, where YL-0919 (mentioned above as a potential sigma-1 receptor ligand) significantly inhibited the excitability of GABAergic neurons in GAD67-GFP transgenic mice. Moreover, the inhibition of GABAergic neurons by YL-0919 was abolished by pretreatment with WAY100635 (Zhang et al., 2021). Although we need to further investigate the specific regulatory mechanisms of this discovery, it further strengthens the idea that the 5-HT system plays a central role in the “antidepressant-like” properties of sigma-1 receptors. This antidepressant mechanism may be involved in the “monoamine(5-HT)-Glu/GABA long neural circuit” (more information is reviewed by Prof. Li (Li, 2020)), and E/I rebalancing should be the critical rate-limiting step for the onset of action. It is important to note that available studies on sigma-1 receptors in relation to the 5-HT system are limited, and still lack direct evidence that sigma-1 receptors regulate 5-HT release in the brain. Moreover, some clues have suggested that sigma-1 receptors play a positive role in regulating the 5-HT system, and many antidepressants are known to act via the sigma-1 receptor pathway, even classical SSRIs with or without affinity for sigma-1 receptors have different pharmacological profiles. In the future, whether sigma-1 receptors can interact directly with the serotonin transporter (SERT), 5HT1A, etc. needs further confirmation (Figure 1). Besides, if sigma-1 receptors are activated by pre-administered sigma-1 receptor agonists, is it possible that there will be a higher affinity with 5HT1A? These considerations may be a breakthrough for clarifying the roles of sigma-1 receptors in the regulatory mechanisms of depression.

The pharmacological antidepressant-like effects of sigma-1 receptor ligands tested in animal models and in human clinical trials showed somewhat useful effects, and sigma-1 receptor ligands seem to be potential psychotherapeutic agents. However, there are no drugs that selectively target sigma-1 receptors on the market at this time, which does not mean that the development of drugs targeting sigma-1 receptors is not promising. In contrast, to clearly define the molecular mechanisms of sigma-1 receptors in depression requires more direct evidence and another major concern is regarding the safety profiles of these potential drugs. In the circumstances of the global COVID-19 pandemic, the “magic molecule” sigma-1 receptor may provide new hope. Sigma-1 receptors play an important role in the replication of SARS-CoV-2 in cells and thus serve as a promising therapeutic target for COVID-19 infections (Hashimoto et al., 2022). In this way, the development of antidepressants based on sigma-1 receptor targets appear to be “game changers” for people with COVID-19, such as the widely available fluvoxamine etc.
